# Divergent short-chain fatty acid production and succession of colonic microbiota arise in fermentation of variously-sized wheat bran fractions

**DOI:** 10.1038/s41598-018-34912-8

**Published:** 2018-11-09

**Authors:** Yunus E. Tuncil, Riya D. Thakkar, Arianna D. Romero Marcia, Bruce R. Hamaker, Stephen R. Lindemann

**Affiliations:** 1 Food Engineering Department, Ordu University, Ordu, 52200 Turkey; 2 Whistler Center for Carbohydrate Research, Department of Food Science, Purdue University, West Lafayette, IN 47907 USA; 3 Department of Food Science and Technology, Universidad Zamorano, El Zamorano, 11101 Honduras; 4 Department of Nutrition Science, Purdue University, West Lafayette, IN 47907 USA

## Abstract

Though the physical structuring of insoluble dietary fiber sources may strongly impact their processing by microbiota in the colon, relatively little mechanistic information exists to explain how these aspects affect microbial fiber fermentation. Here, we hypothesized that wheat bran fractions varying in size would be fermented differently by gut microbiota, which would lead to size-dependent differences in metabolic fate (as short-chain fatty acids; SCFAs) and community structure. To test this hypothesis, we performed an *in vitro* fermentation assay in which wheat bran particles from a single source were separated by sieving into five size fractions and inoculated with fecal microbiota from three healthy donors. SCFA production, measured by gas chromatography, uncovered size fraction-dependent relationships between total SCFAs produced as well as the molar ratios of acetate, propionate, and butyrate. 16S rRNA sequencing revealed that these size-dependent metabolic outcomes were accompanied by the development of divergent microbial community structures. We further linked these distinct results to subtle, size-dependent differences in chemical composition. These results suggest that physical context can drive differences in microbiota composition and function, that fiber-microbiota interaction studies should consider size as a variable, and that manipulating the size of insoluble fiber-containing particles might be used to control gut microbiome composition and metabolic output.

## Introduction

The human colon is one of the most densely-colonized microbial habitats found on earth, being home to tens of trillions of microbial cells^1,2^; these are collectively termed the colonic microbiota. The colonic microbiota and their products are increasingly recognized as being physiologically important for human health^3,4^. Reductions in colonic microbiota diversity have recently been found to correlate with multiple disease states, such as metabolic syndrome and type 2 diabetes^5^, inflammatory bowel disease^6^, and colorectal cancer^7^. Recently, many research groups have determined that (1) this loss of species from the colonic microbiota is linked to consumption of the high-fat, low-fiber Western diet (although differences exist within Western populations with respect to habitual diets (and, consequently, both fiber and other nutrient intake) and gut microbiome diversity^8^) (2) that, in mice, these extinctions compound irrevocably over generations, and (3) that higher consumption of fermentable dietary fibers increases the diversity of the colonic microbiota^9^.The mechanisms driving these losses in diversity remain poorly understood^10^, which inhibits design of dietary strategies to stably increase colonic microbiota diversity as a means to prevent or treat chronic disease.

Dietary fibers are defined as the portion of food, typically of plant origin, that cannot be digested intestinally by human enzymes. Thus, these dietary compounds transit the upper gastrointestinal tract largely intact and reach the colon where, along with host-secreted glycans, they become main energy sources for the microbiota^11–14^. The term *dietary fiber* encompasses (1) non-starch polysaccharides, such as arabinoxylans, pectin, cellulose and gums, (2) resistant oligosaccharides such as fructoologosaccharides and galactooligosaccharides, (3) other digestion-resistant carbohydrates such as resistant starches and dextrins, (4) animal-derived carbohydrates such as chitin, chondroitin sulfate and hyaluronan, and (5) non-carbohydrate-based compounds such as lignin, which is found in plant cell walls^13^. Anaerobic metabolism of dietary fibers by colonic microbiota results in short-chain fatty acids (SCFAs; specifically, acetate, propionate, and butyrate) as the predominant terminal products of fermentation; these SCFAs are increasingly understood to modulate host physiological processes and are thought to contribute to health through multiple mechanisms^15–19^.

Cereal-derived dietary fibers (those from wheat, corn, oat, barley and rye) represent a relatively large fraction of human dietary fiber intake, which is driven in Western countries mainly by wheat consumption^20^. The total dietary fiber content of wheat kernels reaches up to 18% of dry matter^20^, of which the bran portion contains the vast majority^21^. Most of wheat bran dietary fiber is accounted for by non-starch polysaccharides such as arabinoxylans (also known as hemicellulose; ~70%), cellulose (19%), (1–3), and (1–4)-β- glucans (6%)^22^. Other non-starch polysaccharides such as glucomannan, arabinogalactan, and xyloglucan may also be present in small amounts^23^. The majority of the fibers found in wheat bran are water-insoluble due to their interactions with each other and with other cell wall components, such as proteins and phenolic compounds, via covalent bonding, hydrogen bonding and electrostatic interactions^24^.

Recent studies have revealed that wheat bran impacts the structure and metabolic function of the colonic microbiota. For example, wheat bran was shown in an *in vitro* fermentor system to promote butyrate-producing bacteria belonging to family *Lachospiraceae*, which subsequently resulted in an increase in the butyrate concentration^25^. Similar results were also observed in two independent *in vivo* studies, in which consumption of a wheat bran-enriched diet increased butyrate concentrations in the feces of obese^26^ and overweight^27^ humans. These changes were attributed to stimulation of members of *Lachnospiraceae* in the colons of these subjects. Increased butyrate formation in the large intestine through wheat bran fermentation was also reported to suppress chemically-induced tumors in rodents^28,29^.

Though it is well-known that westernization of diet is associated with reductions in total fiber intake, one commonly-overlooked correlate is a decrease in the average particle size of grain flours consumed^30^. It is known that the physical structure of insoluble fiber sources significantly impacts fiber bioavailability and microbial activity^31–34^, yet the size of insoluble fiber particles has not been rigorously examined for its impact upon the gut microbiota. Here, we test the hypothesis that wheat bran fractions varying in size (and, therefore, with different surface area-to-volume ratios) would be fermented differently by gut microbiota, which would lead to size-dependent differences in metabolic fate (measured as short-chain fatty acids; SCFAs) and community structure. Our results, obtained using five size fractions of sieved wheat bran particles, reveal that there is a direct relationship between metabolic outcomes (SCFA production) and wheat bran size fraction. The relative abundances of observed operational taxonomic units (OTUs, surrogates of microbial species) are also directly linked to size fractions. Broadly, these data suggest that controlling the size of insoluble fiber-containing particles might be potentially employed in future dietary strategies to modulate the diversity, composition, and metabolic outcomes (e.g., SCFA production) of the gut microbiome for improved health.

## Results

### Wheat bran particle size strongly influenced the amounts and proportions of SCFAs produced

**Figure 1:**
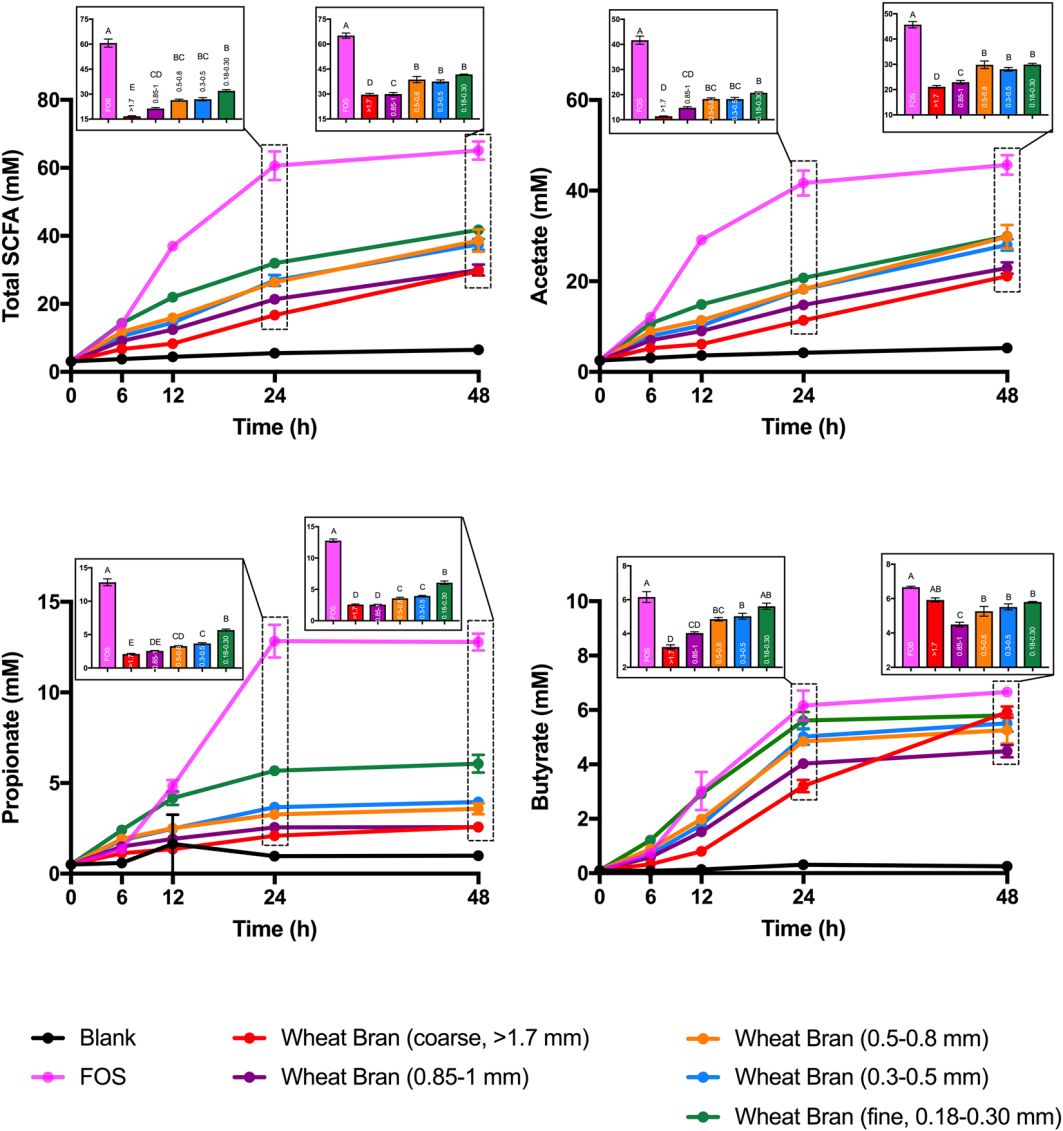
Short-chain fatty acid (SCFA) production by fecal microbiota in *in vitro* fermentations over time. FOS (fructooligosaccharide) was used as a fast-fermenting, butyrate-producing positive control. The blank did not contain any carbon substrate. Total SCFA is the sum of acetate, propionate, and butyrate. Error bars represent the standard error of the mean of three separate replicates. Mean values with the same letter are not significantly different (Tukey’s multiple comparisons test, *p* < 0.05).

**Figure 2:**
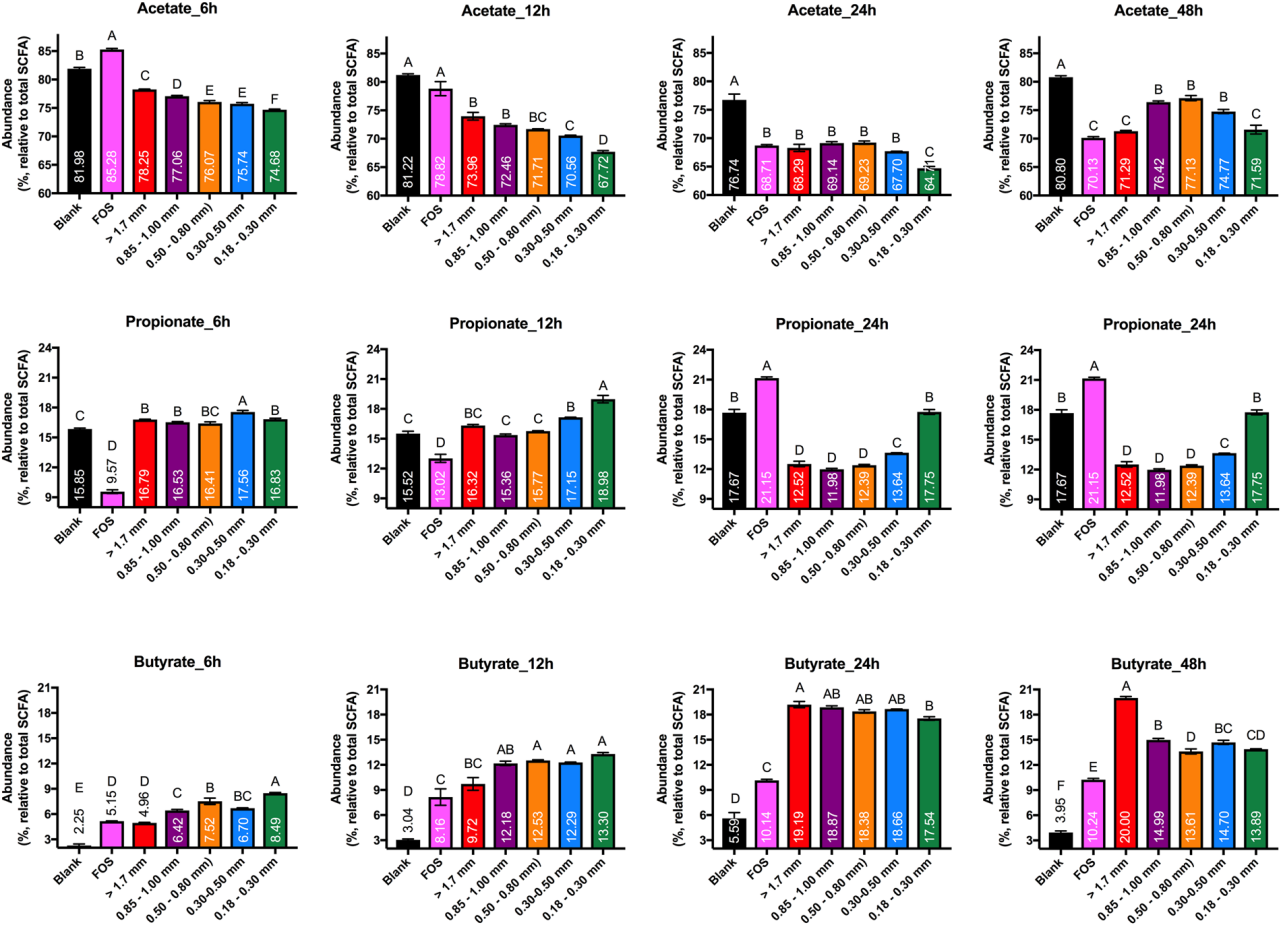
Relative abundances of acetate, propionate and butyrate (relative to total SCFA) produced by fecal microbiota in *in vitro* fermentations over time. FOS (fructooligosaccharide) was used as a fast fermenting-butyrate producing comparator. The blank did not contain any substrate. Error bars represent the standard error of the mean of three separate replicates. Mean values with the same letter are not significantly different (Tukey’s multiple comparisons test, *p* < 0.05). Total SCFA is the sum of acetate, propionate, and butyrate.

We tested whether wheat bran particle size would impact the metabolism and community structure of fecal microbiota through *in vitro* batch fermentations of wheat bran size fractions (separated by sieving from an identical initial source), which had been previously digested enzymatically *in vitro* to mimic transit through the stomach and small intestine and inoculated with fecal microbiota from three healthy donors. To evaluate metabolic outcomes, we measured the production of the terminal SCFAs most abundantly found in fecal samples (acetate, propionate, and butyrate) at 0, 6, 12, 24, and 48 h time points (post-inoculation with fecal microbiota). The particle size fractions tested were (1) 180–300 μm (the finest bran), (2) 300–500 μm, (3) 500–800 μm, (4) 850–1,000 μm, and (5) >1,700 μm (the coarsest bran). The rapidly-fermentable soluble fiber, fructooligosaccharide (FOS)^35^, was also included as a positive control for fermentation. As we hypothesized, different wheat bran size fractions not only impacted the rate and absolute amounts of SCFAs produced by fecal microbiota (Fig. 1) but also significantly (*p* < 0.05) influenced the molar ratios of the SCFAs (Fig. 2). At all time points, fermentation of FOS generated the highest amount of total SCFA, acetate, and propionate (Fig. 1). Among bran fractions, the finest bran produced the highest amount of total SCFA, acetate, and propionate at all the time points, and gradual decreases in the amounts of these microbial byproducts were observed as the particle size increased. The same trend was also observed for butyrate production up to 24 h post-inoculation within the bran fractions. After this time point, fermentation of the finest bran fraction generated butyrate concentrations indistinguishable from FOS, which is widely-regarded as a highly-butyrogenic fiber^35^. Interestingly, although the coarsest bran generated the lowest amount of butyrate in the first 24 h post-inoculation, by 48 h post-inoculation butyrate concentrations in coarse bran fermentations were also indistinguishable from those of FOS (*p* < 0.05) (Fig. 1).

In terms of SCFA molar ratios, at early time points (6 and 12 h post-inoculation) there was a direct relationship between size fraction and the proportion of acetate with respect to the total of all SCFAs (R^2^ = 0.94, and R^2^ = 0.79, respectively; Fig. S1), with the coarsest wheat bran producing the highest proportion of acetate and the finest one generating the lowest proportion (Fig. 2). However, this relationship was not maintained over increasing incubation times (Fig. S1). For propionate generation no significant differences were observed at the 6 h time point between treatments, except that fermentation of 300–500 μm wheat bran particles resulted in the highest propionate production. At later time points (24 h post-inoculation and later), propionate proportions of the two smallest size fractions were significantly higher than the other bran treatment groups but not statistically distinguishable from each other (*p* < 0.05) (Fig. 2). The relationship between particle size fraction and butyrogenesis was temporally complex; we observed an inverse relationship between butyrate molar ratio and wheat bran particle size fraction at early time points (6 and 12 h post-inoculation) with the coarsest wheat bran generating the lowest proportion of butyrate and the finest one the highest (Figs 2 and S1). Surprisingly, this inverse relationship reversed at later time points (48 h), at which a direct relationship between relative abundance of butyrate and wheat bran particle size fraction emerged (R^2^ = 0.84), with the highest proportion of butyrate produced from the coarsest bran (Figs 2 and S1). The relative abundance of butyrate produced from the coarsest bran at 48 h post-inoculation reached 20%, which was almost double the relative abundance of butyrate generated from FOS (10.24%) (Fig. 2). These data clearly indicated that particle size fraction dramatically influenced the metabolic outcome of wheat bran fermentation by fecal microbiota.

### Wheat bran particle size fraction significantly impacted the fecal microbiota community structure

**Figure 3:**
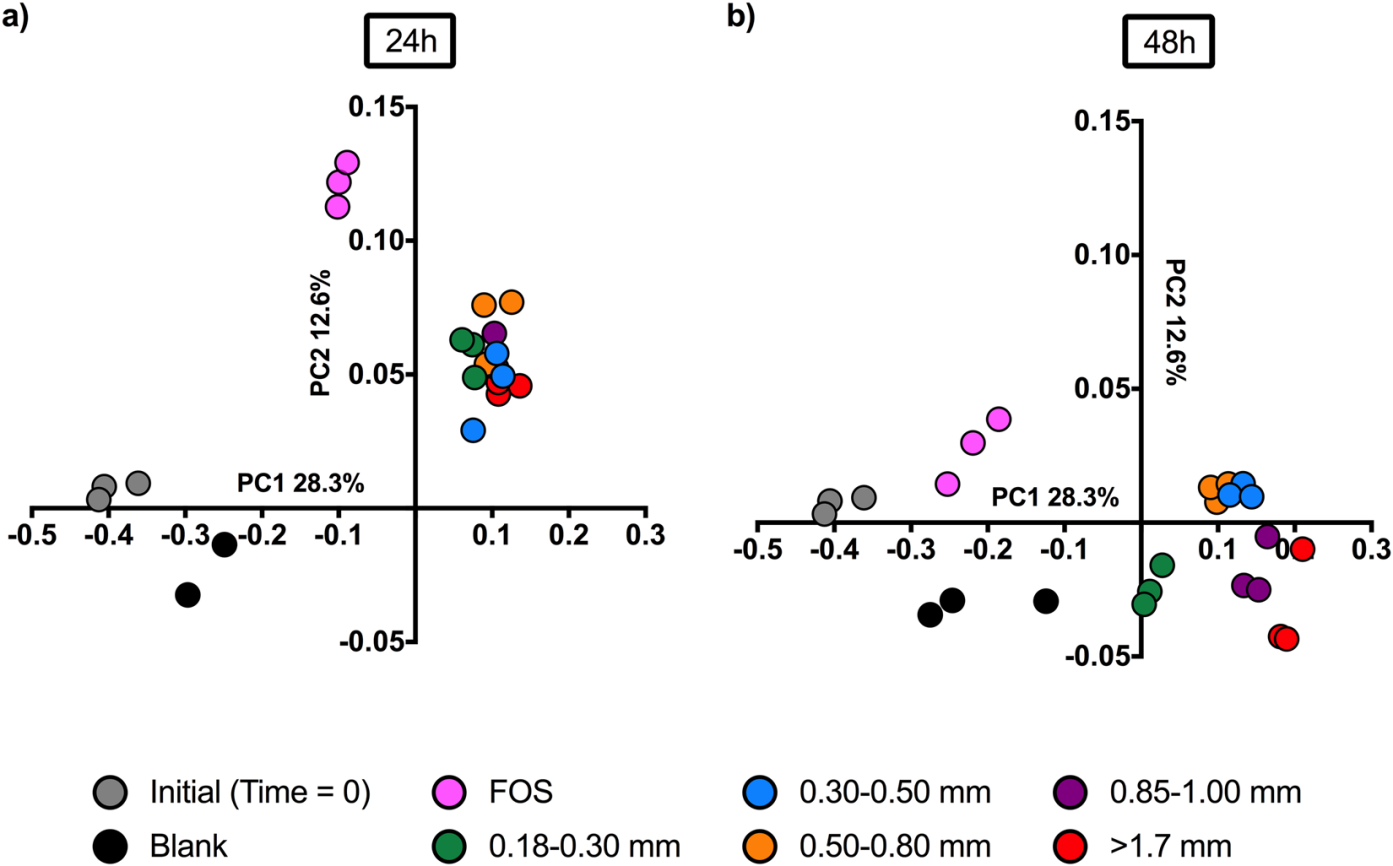
Principal component analysis of community structures associated with wheat bran size fractions, as determined by 16S rRNA gene amplicon sequencing. Bray-Curtis dissimilarity of fecal microbiota was based on the relative abundances of OTUs at a 97% identity level after *in vitro* fermentation for (**a**) 24 h and (**b**) 48 h. FOS (fructooligosaccharide) was used as a fast-fermenting, butyrate-producing positive control. The blank did not contain any substrate. Dissimilarity was also calculated using ThetaYC; the result was not substantially different from that visualized by Bray-Curtis dissimilarity.

To determine whether the observed alterations in metabolism of wheat bran size fractions were accompanied by shifts in the microbiota, we assessed the effects of wheat bran size fraction on colonic microbiota composition by amplicon sequencing targeting the V4 and V5 region of the bacterial 16S rRNA gene, using genomic DNA extracted at 24 and 48 h post-inoculation. Sequences were clustered into OTUs defined at the 97% identity level, from which α- and β-diversity metrics were calculated. As expected, fermentation of FOS and bran fractions resulted in very different microbial community structures over time (Fig. 3). Within bran-consuming cultures, the microbiota associated with distinct size fractions were significantly different across both time points (AMOVA, *p* < 0.001), although we observed no significant differences (*p* < 0.05) in α-diversity metrics (Fig. S2). Although size fraction clusters were not clearly resolved after 24 h post-inoculation, (Fig. 3a), clear separations between microbiota consuming different bran size fractions were evident 48 h post-inoculation. Especially evident were demarcations of microbiota growing on the finest wheat bran treatment and those consuming the coarsest bran (Fig. 3b). Taken together, these data suggest that divergent communities arise as colonic microbiota ferment wheat bran particles of differing size fractions.

### The selective effect of bran particle size fraction operates at fine taxonomic resolutions

**Figure 4:**
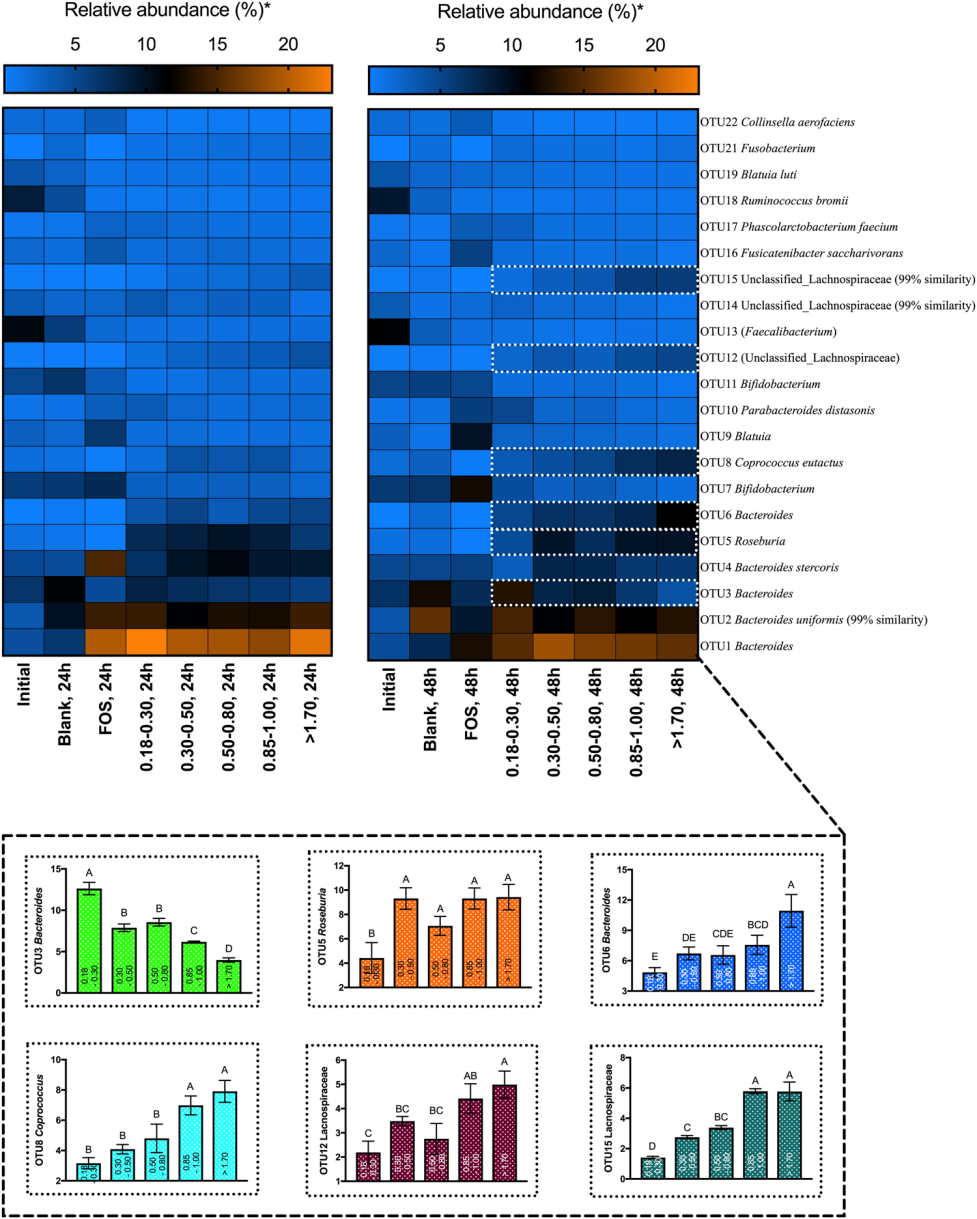
Relative abundances (percentage of sequences) based on the top 50 OTUs in each sample. The top 50 OTUs account for more than 90% of the total sequences of all wheat bran treatment groups at all time points (Fig. S7). Error bars represent the standard error of the mean of three separate replicates. Mean values with the same letter are not significantly different (Tukey’s multiple comparisons test, *p* < 0.05).

Though particle size fraction resulted in significant differences in abundances of taxa at the family level and higher (Fig. S3), the selective effects of particle size also occurred at the genus or species levels. In general, overrepresentation of phylum *Firmicutes* in association with the coarsest brans was driven by increases in members of *Lachnospiraceae*, whereas increases in the relative abundances of members of *Bacteroidaceae* drove increased representation of phylum *Bacteroidetes* associated with the finest brans. However, within genus *Bacteroides*, distinct OTUs increased in abundance in response to differential bran size fractions. The most obvious change within bran treatments, compared to the inoculum, was a 20-fold increase in the relative abundance of OTU6 *Bacteroides* (of which 59% of reads within the cluster could be classified as *B. intestinalis* with a bootstrap value ≥95%) with the coarsest bran treatment. Similarly, we observed 4-, 8-, 20-, and 23-fold increases over the initial microbiota in the relative abundances of OTU8 *Coprococcus eutactus*, OTU5 *Roseburia*, OTU12 *Lachnospiraceae* and OTU15 *Lachnospiraceae*, respectively, in fermentations of the coarsest bran. In contrast, growth on wheat bran caused dramatic decreases in the relative abundances of *Bifidobacterium*-related OTUs; the coarsest wheat bran treatment resulted in 5-, and 3-fold decreases in OTU11, and OTU7, respectively, compared to the inoculum (Figs 4 and S4).

More surprisingly, we observed either direct or inverse relationships between individual OTU abundances and wheat bran size fraction. For example, there was an inverse relationship between OTU3 *Bacteroides* (of which 67% of reads could be classified within *B. dorei*) and wheat bran particle size fraction (R^2^ = 0.775) (Fig. S5), such that increasing particle size led to decreasing relative abundance of this OTU (Figs 4, S4 and S5). Conversely, at the 48 h post-inoculation time point, OTU6 *Bacteroides* (of which 59% of the reads could be classified within *B. intestinalis*), OTU8 *Coprococcus eutactus*, OTU12 *Lachnospiraceae* and OTU15 *Lachnospiraceae* displayed a direct relationship with wheat bran size fraction (R^2^ = 0.95, R^2^ = 0.89, R^2^ = 0.76, R^2^ = 0.76, respectively) such that the relative abundances of these species gradually increased with increasing wheat bran particle size (Figs 4, S4 and S5).

**Figure 5:**
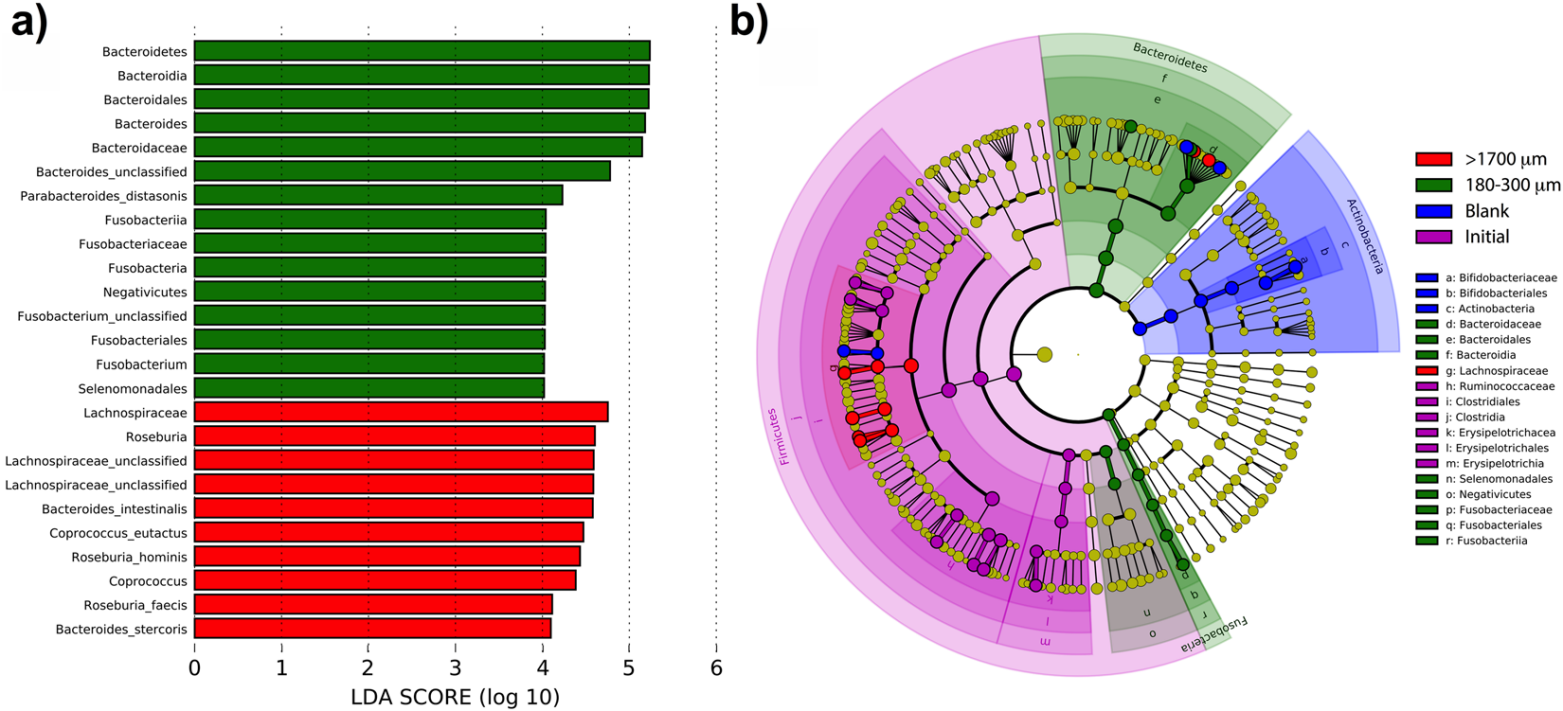
Linear discriminant analysis of taxa differentiating the finest and coarsest wheat bran size fractions. Initial and blank communities were also included in the analysis to prevent misattribution of taxa to size fractions that were more highly represented in controls. (**a**) Taxa with LDA scores >4.0 in the finest (180–300 μm) and coarsest (>1700 μm) size fractions at 48 h post-inoculation; linear discriminants of initial and blank conditions not shown. (**b**) Cladogram depicting taxa that are overrepresented in the finest and coarsest bran fractions compared with abundances in the initial inoculum and substrate-free blank incubations.

To identify the specific bacterial taxa representative of the extremes in wheat bran size fractions, we compared the microbial compositions of the coarsest and the finest wheat bran treatments at 48 h post-inoculation using the linear discriminant analysis effect size (LEfSe) method (Fig. 5). Members of *Lachnospiraceae* (specifically, *Coprococcus eutactus* and *Roseburia hominis*) were shown to be discriminators for the coarsest bran treatment, whereas members of *Bacteroides* and its parent taxa were differentiators for the finest bran treatment (Fig. 5a, LDA > 4). However, within genus *Bacteroides*, OTU6 (attributed as *Bacteroides intestinalis*) and OTU4 (attributed as *Bacteroides stercoris*) were differentiators for the coarsest bran particles. These relationships suggest that stable relationships exist between bran size fraction and individual species (especially, members of *Bacteroide*s) within the colonic microbiota.

### Different wheat bran fractions possess distinct monosaccharide compositions

**Figure 6:**
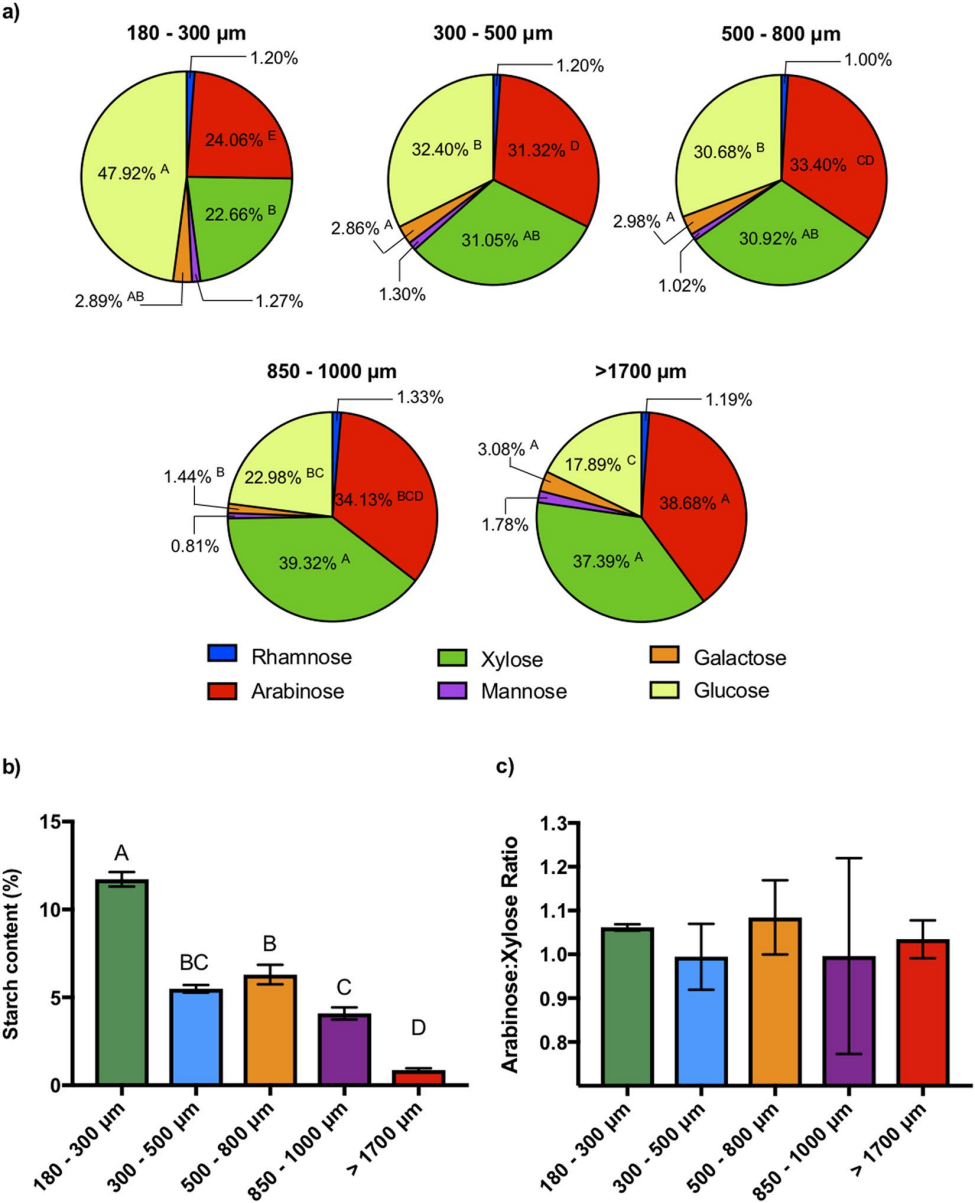
(**a**) Neutral monosaccharide compositions of the wheat bran samples (%, mole basis). Mean values of each constituents were compared across the samples, and those with the same letter are not significantly different (Tukey’s test, *p* < 0.05). No letter was included where the mean values are not statistically different (Tukey’s test, *p* < 0.05). (**b**) Total starch contents of the samples. Error bars represent the standard error of the mean of three separate replicates. Mean values with the same letter are not significantly different (Tukey’s multiple comparisons test, *p* < 0.05). (**c**) Arabinose:xylose ratio of the samples. Error bars represent the standard error of the mean of three separate replicates. No statistical differences were observed between the mean values (Tukey’s multiple comparisons test, *p* < 0.05).

To identify potential mechanisms driving differences in microbial metabolism of wheat bran size fractions and its consequent impact upon community structure, we next investigated whether, in addition to differences in physical size, the variously-sized wheat bran fractions were chemically distinct. Accordingly, we measured the neutral sugar contents of wheat bran fractions after they had been digested with salivary α-amylase, pepsin, and pancreatin enzymes to mimic upper gastrointestinal (GI) tract transit; this treatment removes the vast majority of accessible starch (Fig. 6). No significant differences in the proportions of rhamnose, mannose, and galactose were observed among the samples (*p* < 0.05) (Fig. 6a). The finest wheat bran particles contained the highest amount of glucose (47.92%), and the glucose proportion of the brans decreased as the particle size increased (Fig. 6a). In wheat, the two main sources of glucose are starch and cellulose, so the majority of the glucose measured in the neutral sugar analysis likely belonged to one of these two polysaccharides. We then measured the total starch content of the samples to determine whether the observed glucose proportions correlated with particle starch content (Fig. 6b). Indeed, the finest bran particles displayed the greatest amount of starch (11.72%) and, as particle size increased, starch content decreased (with the coarsest bran displaying less than 1% starch). These data strongly suggested that the distinctions in the glucose proportions measured by neutral sugar analysis arose from differences in the starch, rather than the cellulose, contents of the wheat bran fractions. However, these starches were not removed by treatment with upper-GI-simulating amylase treatment; thus, the starches measured are likely to be resistant.

We also found significant differences in the proportions of xylose and arabinose among the size fractions (*p* < 0.05). In contrast to glucose, we observed direct relationships between wheat bran size fraction and arabinose (R^2^ = 0.83) and xylose contents (R^2^ = 0.77), with the finest bran fraction displaying the lowest proportions of xylose (22.66%) and arabinose (24.06%) (Figs 6a and S6). The relative proportions of these sugars increased with bran size fraction, with the coarsest bran possessing the highest proportions (37.39%, and 38.68% xylose and arabinose, respectively) (Figs 6a and S6). Xylose and arabinose are the building blocks of arabinoxylan polymers, with the former composing the backbone of the molecule and the latter forming the branching points^36^. Therefore, the arabinose-to-xylose ratio is useful in estimating the branch density of the molecule. The arabinose to xylose ratio among bran size fractions were not significantly different (*p* < 0.05) (Fig. 6c), suggesting similar arabinoxylan structure among size fractions.

## Discussion

Consumption of specific dietary fibers has been shown to exert significant selective effects on bacterial taxa in the colon due to variations in genetically encoded fiber-utilization abilities of different species^11,14,37,38^. These selective effects suggest that modulation of the gut microbiota’s structure or function through variation in the type or quantity of dietary fibers consumed may be an effective strategy for countering the rising burden of chronic disease, which is increasingly recognized to be connected to the gut microbiome^39,40^. However, design of such strategies requires a mechanistic understanding of the interactions between dietary fiber types, (along with their associated physical and chemical structural variables) and the colonic microbiota, as subtle variations in carbohydrate structure are known to dramatically influence microbial responses to the substrate^13,41,42^. Many studies have been conducted to determine how variations in the chemical structures of dietary fibers impact the responses of individual bacterial species^43^ as well as overall colonic microbiota composition^36,44,45^. In contrast, studies focusing on interactions between physical structural variables (e.g., particle size) of insoluble fibers and colonic microbiota have heretofore been relatively sparing.

Here, we show that wheat bran particle size fraction, a component of insoluble fiber physical structure, strongly influences both the metabolism and composition of gut microbiota in *in vitro* fermentations. Alterations in community structure were evident at the family level, with coarser size fractions favoring members of *Lachnospiraceae* and finer fractions generally favoring members of *Bacteroidaceae* (Fig. S3b). Within these trends at higher taxonomic levels, however, differential responses to size fractions revealed competition at the species or strain level within genus *Bacteroides*. In some cases, OTU relative abundances displayed nearly-linear relationships with fraction size, with the relative abundance of *Bacteroides* OTUs generally decreasing as size increases. However, the effect was species-specific, as *Bacteroides* OTU6 increased alongside *Coprococcus* and unclassified *Lachnospiraceae* OTUs with size (Figs 4, S4, and S5).

Even though members of *Bacteroides* members are typically regarded as generalists that can digest a broad range of plant cell-wall polysaccharides^14,46–48^, they seem to be better adapted to the degradation of soluble fibers compared to insoluble substrates, including wheat bran particles^49–51^. Only one cellulolytic *Bacteriodes* sp., *B. cellulosilyticus*, has been described to date, suggesting the ability to degrade microcrystalline cellulose-rich cell wall structures may be rare within the genus^52^. In contrast, many members of *Lachnospiraceae* are widely reported to be fibrolytic and, along with the some members of neighboring family *Ruminococcaceae*, play an important role in degradation of plant cell wall polysaccharides, including cellulose^53^. These microbial taxa ferment cell wall polysaccharides and starches to butyrate^54,55^. As members of *Bacteroides* are more commonly propiogenic than butyrogenic^56^, the abundance of *Bacteroides* in association with fine particle fractions (Figs 4, S4, and S5) may explain elevated propionate molar ratios in these cultures (Fig. 2).

Another interesting finding from this study is that the coarsest bran was as butyrogenic as FOS, and this butyrate formation occurred slowly. This further suggests that coarser wheat bran particles may deliver more butyrate into more distal regions of the colon than finer ones. Thus, coarse wheat bran particles could have important practical applications, as the majority of chronic colonic diseases, such as ulcerative colitis, inflammatory bowel diseases, and colorectal cancer occur in the more distal regions of the colon^57–59^ and butyrate has been implicated as important for mitigating these disease conditions^60,61^.

Though previous studies have detected differences in SCFA production from different wheat bran size fractions^32,33^, this study holistically links size-dependent metabolic outcomes with underlying microbial community structures. Our results agree with one previous study investigating SCFA production from fine and coarse wheat bran size fractions, which reported that fermentation of coarse wheat bran particles (with an average particle size of 1239 µm) by fecal microbiota resulted in higher butyrate production compared to fine wheat bran fractions (with an average particle size of 551 µm) 24 h post-inoculation^32^. However, in the experiment of Dziedzic and coworkers, fine wheat bran (average particle size ~90 µm) produced more butyrate than coarse bran (average particle size ~500 µm), though the incubation time was not reported^33^. In addition to differences in the particle sizes investigated, disagreement in the results may also stem from distinct fermenting bacteria; Dziedzic and coworkers use cultivation-based detection methods and report only the abundances of *Enterococcus*, *Bifidobacterium*, *Escherichia coli*, and *Lactobacillus*. It is unclear whether other species were present in the inoculum, as the organisms were described as fecal isolates. In a more recent *in vivo* study, where mice were fed a Western-simulating diet supplemented with either coarse (particle size: 1690 µm) or fine (particle size: 150 µm), similar in size range to that examined in the present study, wheat bran indicated no significant changes in the SCFA levels in the cecum of the mice; however, coarse (but not fine) bran fractions promoted fat excretion and increases in the abundance of genus *Akkermansia*^34^. Though these data hint that different bran size fractions may exert distinct physiological impacts *in vivo*, previous studies have not investigated this effect at high size resolution. Near-linear relationships between microbial metabolism and community structure detected in the present study suggest that increasing size resolution may help tease out *in vivo* relationships between bran fraction size, microbiota, and host physiological outcomes, as the number of potentially-confounding variables is much greater *in vivo*.

Importantly, this study reveals differences in the chemical structures of wheat bran size fractions (Fig. 6), even though these brans were fractionated by directly sieving the wheat bran obtained from the same milling process. This observation has two important implications. The first is that variation in chemical structures might be the driving force for the formation of different particle sizes during the milling process. This leads naturally to the second implication, that these chemically-distinct size fractions do not perform identically in their interaction with the gut microbiota. Taken together, these data suggest that future studies involving the interaction of wheat brans (and possibly other insoluble fiber sources) with the gut microbiota both *in vitro* and *in vivo* should measure and report physical characteristics – such as particle size – of the bran sources employed. Unreported differences in size fractions may otherwise drive inconclusive or confusing results due to differences in physical structure, chemical composition, and/or fermentation by the microbiota.

In a broader sense, our data support the idea that physical structuring of insoluble particles may lead to significant differences in spatial patterning of substrates at relatively-fine spatial scales (on the order of hundreds of microns), which in turn leads to alternate microbial succession as substrates for some organisms are made available due to the metabolism of others. Although it should be noted that our study was limited to three donors and individuality effects may impact microbial bran fermentation^62,63^, our results suggest that bran particles may generally function ecologically as patchy habitats in which heterogeneous patterning of resources^64^ drives microbial metabolism and community structure outcomes. However, it further suggests that differences in these spatial patterns give rise to temporal patterns in resource availability, from which distinct succession and trophic networks arise through interactions among organisms in particle-degrading consortia^65^. We submit that future studies should (1) focus on isolation of bran particle size as a variable distinct from chemical composition (if possible), (2) advance mechanistic understanding of bran particle substrate distribution and fermentation by gut microbiota, and (3) identify how interactions among organisms interface with the physical structure of bran particles to affect the succession of fermenting microbiota.

## Materials and Methods

### Wheat brans

Wheat bran were a generous gift of The Mennel Milling Company (Fostoria, OH). Different size fractions of wheat brans were obtained by sieving the wheat bran through various screens (sizes 180, 300, 500, 800, 850, 1000, and 1700 μm) using a sieving machine (Portable Sieve Shaker Model RX-24, sieving machine and screens both from W.S. Tyler Combustion Engineering, Inc., Mentor, OH). Before analyses, wheat bran fractions were treated with alpha-amylase, pepsin and pancreatin enzymes *in vitro* to simulate digestion through the upper gastrointestinal tract digestion, followed by dialysis (3.5 kDa cutoff; Spectra/Por 3, Spectrum Labs, Rancho Dominguez, CA) for 36 hrs and lyophilization, as previously described^35,66,67^. This dialysis step mimics absorption process occurring in the small intestine by removing all the small compounds, including simple sugars, generated during enzyme treatments.

### Sugar composition analysis

Neutral monosaccharide composition of the digested wheat brans were determined as their alditol acetate derivatives using separation by gas chromatography on a capillary column (SP2330; SUPELCO, Bellefonte, PA) coupled with mass spectrometry (GC/MS; models 7890A and 5975C inert MSD with a Triple-Axis detector, Agilent Technologies, Inc., Santa Clara, CA), as previously described^68^. Helium was used as a carrier gas. The GC/MS run conditions are as follows: injection volume of 2 µl with a split ratio of 1:2; injector temperature at 240 °C; detector temperature at 300 °C; the gradient temperature program set was 160 °C for 6 min, then 4 °C /min to 220 °C for 4 min, then 3 °C /min to 240 °C for 5 min, and then 11 °C /min to 255 °C for 5 min.

The total starch contents of the samples were determined using a total starch assay kit (Product Code: K-TSTA) according to the manufacturer instructions (Megazyme International, Wicklow, Ireland).

### *In vitro* fermentation

*In vitro* fermentation assays were performed in triplicate as previously described^35,69^ in an anaerobic chamber (BACTRONEX Anaerobic Chamber; Shel Lab, Cornelius, OR) under an 85% N_2_, 5% CO_2_, and 10% H_2_ atmosphere. Briefly, carbonate-phosphate buffer was prepared and sterilized by autoclaving at 121 °C for 20 min. The buffer was then cooled to room temperature, oxygen was removed by bubbling with carbon dioxide, and cysteine hydrochloride (0.25 g/L of buffer) was added as a reducing agent. The buffer was then immediately placed into the anaerobic chamber overnight. Wheat brans (50 mg) were weighed into 25 mL Balch tubes (Chemglass Life Sciences, Vineland, NJ) for each time point (0, 6, 12, 24 and 48 h). Tubes containing the substrates were autoclaved and then transferred into the anaerobic chamber.

The following day, 4 ml of carbonate-phosphate buffer was added to each Balch tube. Fecal samples were collected from three healthy donors who were consuming their routine diets and had not taken antibiotics for at least 3 months. Two of the donors were female (one 23-year-old vegetarian and 28-year-old omnivore) and the other was a 27-year-old omnivorous male, all of whom had normal BMI (18.5 kg/m^2^ < BMI < 25 kg/m^2^). Fecal samples were tightly sealed in plastic tubes, kept on ice prior to rapidly being transferred into the anaerobic chamber, and used within 2 h of collection. The fecal samples were homogenized with carbonate-phosphate buffer [feces:buffer 1:3 (w/v)], followed by filtration through four layers of cheese cloth. Filtered fecal slurries were pooled, and then 1 ml of pooled fecal slurry was inoculated into each tube. Pooling fecal slurries is a common method to investigate how variations in the fine structures of dietary components impact the colonic microbiota^36,67,70–72^, providing a diverse initial pool of species that is not constrained by the idiosyncratic gut microbiomes of individuals. Use of pools of fecal microbiota for *in vitro* studies results in similar microbial community profiles and activities compared to those obtained from single donors^73^. The tubes were then immediately closed with butyl rubber stoppers (Chemglass Life Sciences), sealed with aluminum seals (Chemglass Life Sciences), and incubated at 37 °C in a shaking water bath (150 rpm). Test tubes containing no substrate and FOS (50 mg, Sigma-Aldrich) were used as a blank and fast-fermenting fiber control, respectively, at each time point. All analyses were performed in triplicate. Protocols involving human stool collection and use were reviewed and approved by Purdue University’s Institutional Review Board (IRB protocol #1701018645), all methods were carried out in accordance with the IRB protocol, and all participants provided informed consent.

### Total gas production and sample collection for SCFA and DNA analysis

At each time point, total gas production was measured with a graduated syringe by passing the needle through the rubber stopper (Fig. S8), and the tubes were then opened. Two aliquots were collected from each tube for DNA extraction (1 ml) and SCFA analysis (0.4 ml). Samples collected for DNA extraction were immediately stored at −80 °C until further analysis. 100 ml of an internal standard mixture (prepared by combining 157.5 µl of 4-methylvaleric acid, 1.47 ml of 85% phosphoric acid, 39 mg of copper sulfate pentahydrate in a final volume of 25 ml ultrapure water) were immediately added to samples collected for SCFA analysis, which were then vortexed and stored at −80 °C until analysis.

### SCFA analysis

SCFA analyses were performed as previously described^35^. Briefly, the samples were thawed at room temperature and centrifuged at 13,000 rpm for 10 mins. Supernatants (4 µl) were analyzed using a gas chromatography (GC-FID 7890 A; Agilent Technologies Inc.) on a fused silica capillary column (Nukon^TM^ SUPELCO No: 40369-03A, Bellefonte, PA) under the following conditions: Injector temperature at 230 °C; initial oven temperature at 100 °C; temperature increase of 8 °C/min to 200 °C with a hold for 3 min at final temperature. Helium was used as a carrier gas at 0.75 ml/min. Acetate (catalog number: A38S), propionate (catalog number: A258), and butyrate (catalog number: AC108111000) purchased from Fisher Scientific (Hampton, NH) were used as external standards. 4-methylvaleric acid (catalog number: AAA1540506, Fisher Scientific) was used as an internal standard for quantification.

### DNA extraction

DNA extraction from the samples were carried out using FastDNA SPIN® kit for Feces (product code: 116570200) according to the manufacturer’s instructions (MP Biomedical, Santa Ana, USA) with the following modification. Samples stored for DNA extraction were centrifuged at 13,000 rpm for 10 mins, and the supernatants were discarded. Pellets were homogenized with phosphate buffer and transferred into the Lysing Matrix E tube. Subsequently, the rest of the isolation was performed according to the manufacturer’s instructions.

### 16S rRNA sequencing

The V4–V5 region of the 16S rRNA gene was amplified by PCR using the universal bacterial primers: 515-FB (GTGYCAGCMGCCGCGGTAA) and 926-R (CCGYCAATTYMTTTRAGTTT)^71^. The PCR solution included 2 µl of template DNA (containing >5 ng/µl DNA), 0.625 µmole of each primer, 12.5 µl of HiFi hot start ready mix (catalog number: KK2602, Kapa Biosystems, Wilmington, MA), and 0.016 µg of BSA (catalog number: BP9706100, Fisher Scientific), in a buffer containing Tris-HCl (final concentration in the reaction 0.8 mM; catalog number: BP1758, Fisher Scientific), KCl (final concentration 4 mM; catalog number: P217, Fisher Scientific), EDTA (4 µM final concentration; catalog number: S311, Fisher Scientific) and glycerol (final concentration 1.6%, catalog number: G33, Fisher Scientific) in a final reaction volume of 25 µL. The cycling parameters were comprised of an initial denaturation at 98 °C for 3 minutes, followed by 22 cycles of denaturation at 98 °C for 10 seconds, annealing at 50 °C for 30 seconds and extension at 72 °C for 30 seconds. Final extension was performed at 72 °C for 10 minutes, after which products were held at 4 °C. Unincorporated dNTPs and primers were removed using the Axygen AxyPrep PCR Clean-up Kit (Axygen Scientific, an imprint of Corning Life Sciences, Tewksbury, MA) according to the manufacturer’s instructions. PCR products were barcoded using the TruSeq dual-index approach, purified again using the AxyPrep PCR Clean-up Kit, quantitated via Qubit dsDNA HS Assay Kit (Invitrogen, Carlsbad, CA), and pooled. Quality control for pools was performed by running 1 µL of each pool on an Agilent Bioanalyzer with a High Sensitivity Chip (Agilent, Santa Clara, CA) followed by quantitation for pool loading via the KAPA Library Quantification Kit for Illumina Platforms. Sequencing was performed using on an Illumina MiSeq run with 2 × 250 cycles and V2 chemistry (Illumina, Inc., San Diego, CA) at the Purdue Genomics Core Facility.

### Sequence processing and community analysis

Sequences were processed using mothur v.1.39.3 according to the MiSeq SOP (https://www.mothur.org/wiki/MiSeq_SOP [accessed 9/6/2017])^74,75^ with the following modifications. Contigs were assembled and assigned to groups permitting no errors within the primer region. Sequences were initially screened for a maximum length of 411 nt, zero maximum ambiguous bases and a maximum homopolymer length of 9 nt, then aligned to the mothur-formatted SILVA reference alignment^76^ across positions 11894 to 27656. Sequences were classified using the mothur-formatted version 16 of the Ribosomal Database Project^77^ training set, to which species epithets had been added to the reference taxonomy using a custom Perl script. Sequences were classified to the species level, where possible, at a bootstrap cutoff of 95% to restrict classification to only very high-confidence classifications. Sequences classified within domain *Eukarya*, as chloroplasts or mitochondrial sequences, or with unknown classification at the domain level were removed from further processing. OTU classifications at the species level are reported as the percentage of reads that were classified within a given species at a bootstrap value of 95% or greater. Ecological α-diversity metrics were calculated using the nseqs, coverage, invsimpson, simpsoneven, chao, and shannon calculators and β-diversity metrics were calculated using the braycurtis and thetayc calculators as implemented in mothur. Distance matrices based upon β-diversity metrics were plotted for visualization using the pcoa command in mothur^75^; the Bray-Curtis^78^ - and the Yue and Clayton^79^-derived principle component analysis plots were very similar in structure. Analysis of molecular variance (AMOVA) tests were also computed between size fractions to determine whether centroids were significantly distinct using the amova command in mothur^75,80–82^. LEfSe-formatted files were generated using mothur using make.lefse, and linear discriminant analysis was performed using LEfSe v.1.6^83^.

### Statistical analyses

All analyses were performed in triplicate. Data are presented as mean ± SEM. Statistical analyses were performed using GraphPad Prism version 7.0 for Mac OS X (GraphPad Software, Inc. La Jolla, CA). Analysis of variance (ANOVA) was performed at α = 0.05 significance level to determine differences among the samples and controls. Tukey’s multiple comparison test at α = 0.05 was used to see whether mean differences were statistically different. Linear regression model was computed using Prism version 7.0 to see the relationship between wheat bran particle size and metabolic outcomes (SCFAs, and OTU abundances).

## Electronic supplementary material

Supplementary Information

## Data Availability

MiSeq reads described in this paper are associated with BioProject PRJNA432190 and have been deposited in GenBank’s Sequence Read Archive as under SRA accession SRP131751.
