# Supplementary material for: Divergent short-chain fatty acid production and succession of colonic microbiota arise in fermentation of variously-sized wheat bran fractions
Source: Sci Rep. 2018 Nov 9;8:16655. doi: 10.1038/s41598-018-34912-8 (PMC6226458; doi:10.1038/s41598-018-34912-8)
Supplement: Supplementary file 1 — Supplementary Information [file 41598_2018_34912_MOESM1_ESM.pdf]

Supplementary Information

Divergent short-chain fatty acid production and succession of colonic microbiota arise in fermentation of variously-sized wheat bran fractions

Yunus E. Tuncil[1,2], Riya D. Thakkar[2], Arianna D. Romero Marcia[2,3], Bruce R. Hamaker[2], Stephen R. Lindemann[2,4]*

[1] *Food Engineering Department, Ordu University, Ordu, 52200, Turkey*

[2] *Whistler Center for Carbohydrate Research, Department of Food Science, Purdue University, West Lafayette, IN 47907 USA*

[3] *Department of Food Science and Technology, Universidad Zamorano, El Zamorano, 11101 Honduras*

[4] *Department of Nutrition Science, Purdue University, West Lafayette, IN 47907 USA*

* Author for correspondence. Email: lindemann@purdue.edu

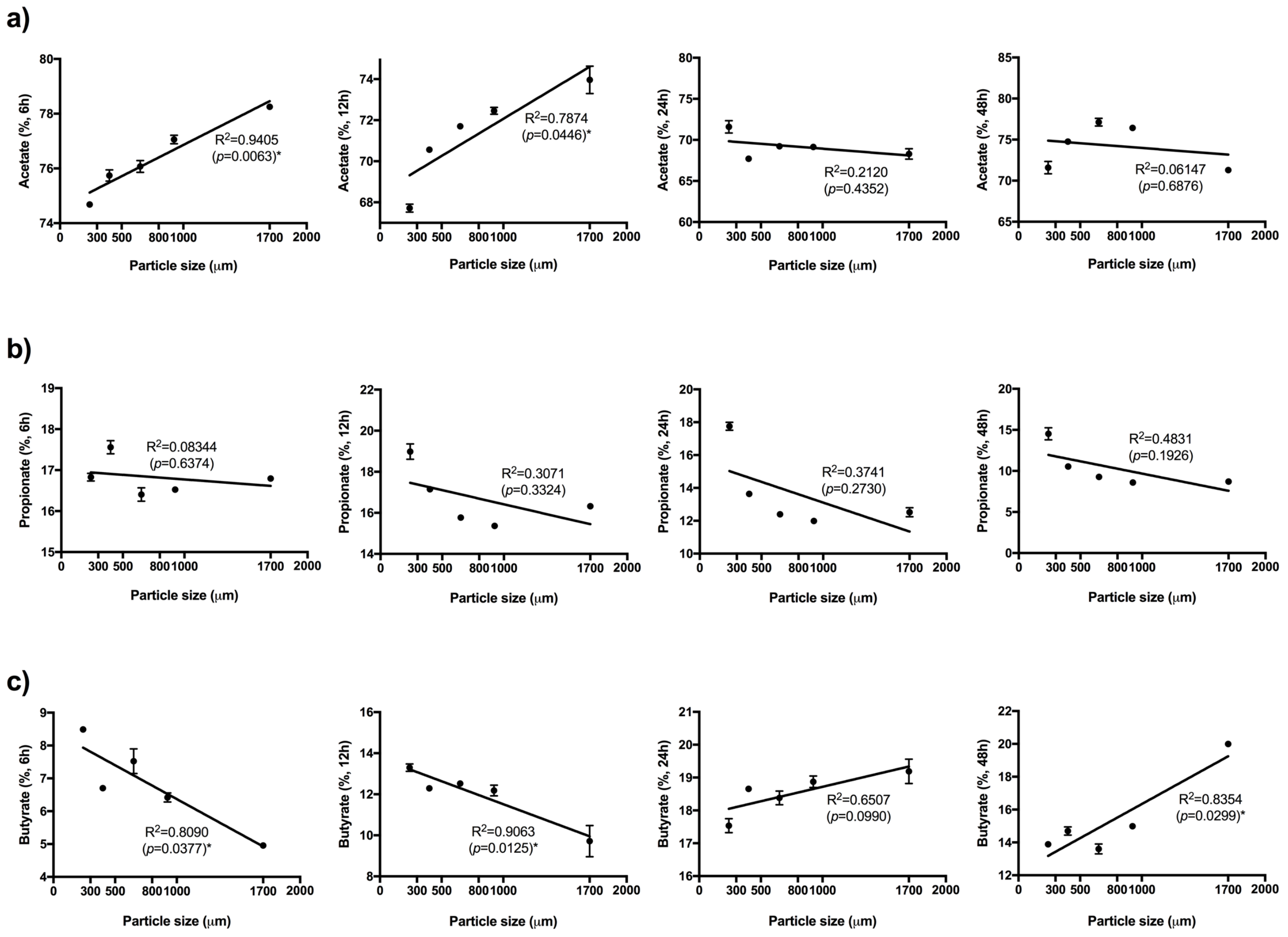

Figure S1. Regression analysis between short chain fatty acid proportions and wheat bran particle size. Average particle size values of each wheat bran treatment groups were taken into account for regression analysis. For example, wheat bran particles between 180-300 μm in size were considered as 240 [(180+300) ÷ 2] μm for the regression analysis. The sizes of brans having >1700 μm particle size were considered as 1700 μm. The regression results having statistical significance (α=0.05) are shown with an asterisk.

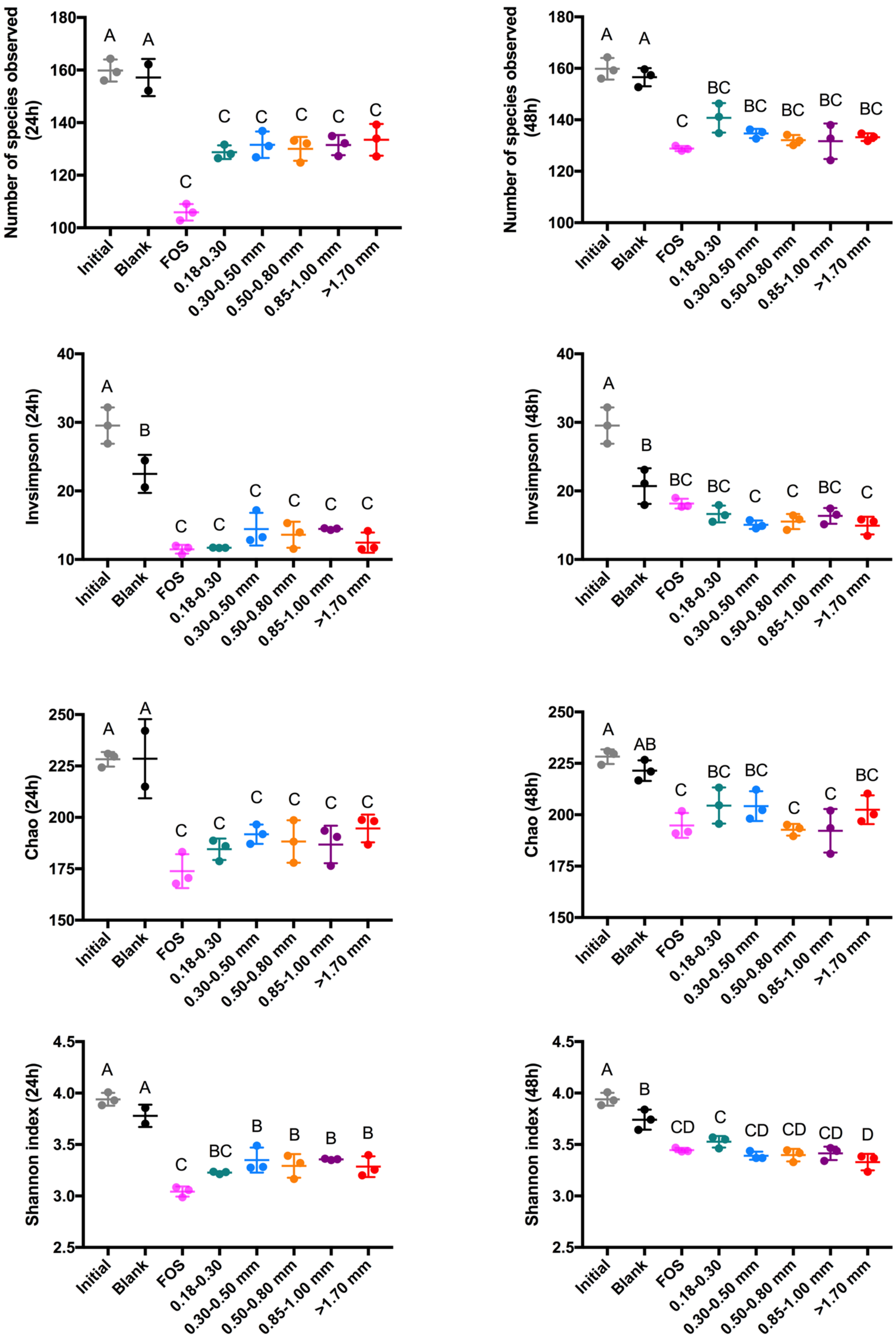

Figure S2. α-diversity analyses of fecal microbial communities throughout *in vitro* fermentation. (Tukey's multiple comparisons test, $p < 0.05$).

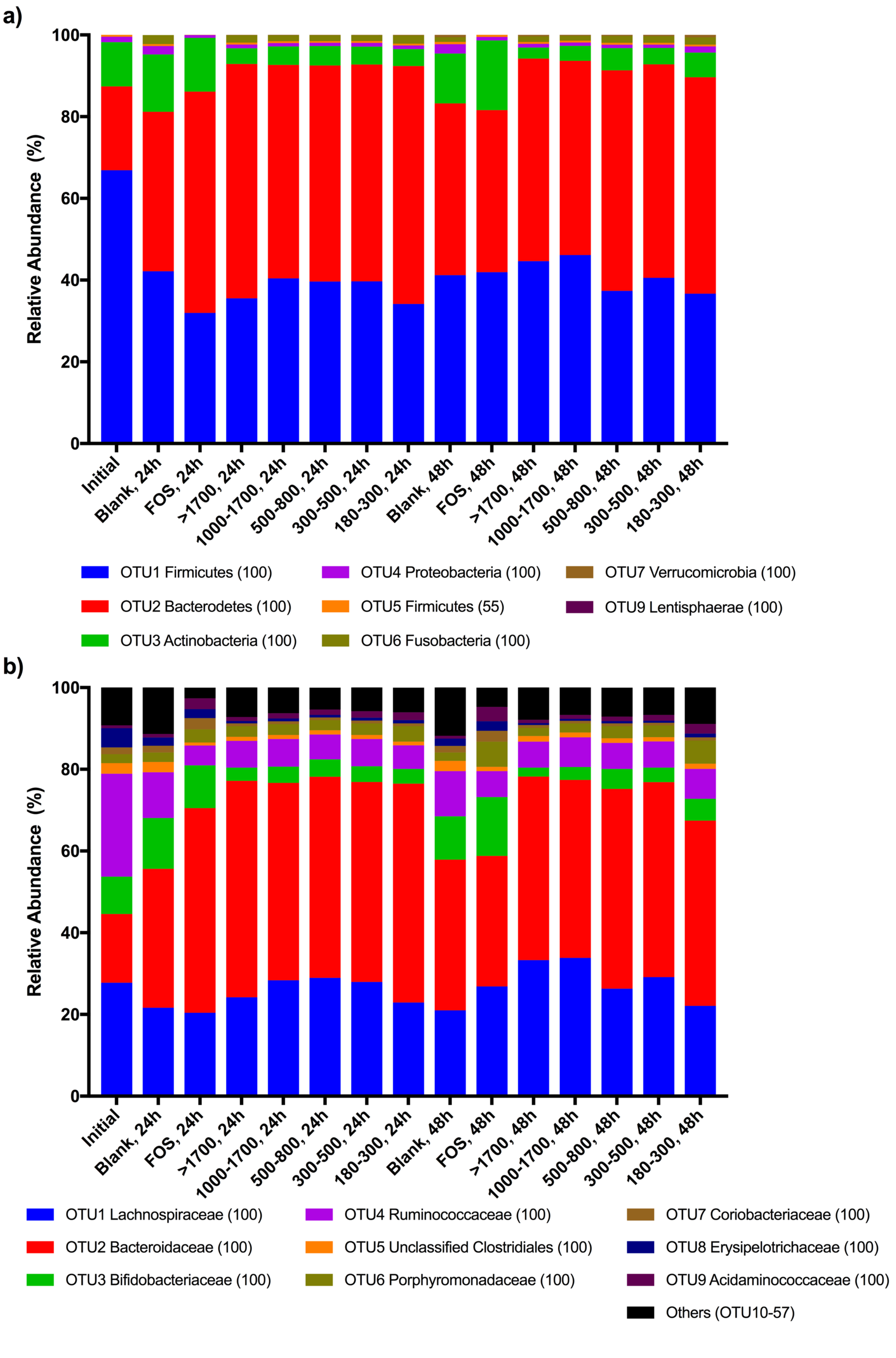

Figure S3. a) Phylum and **b)** family level changes in microbiota composition after the *in vitro* fermentation.

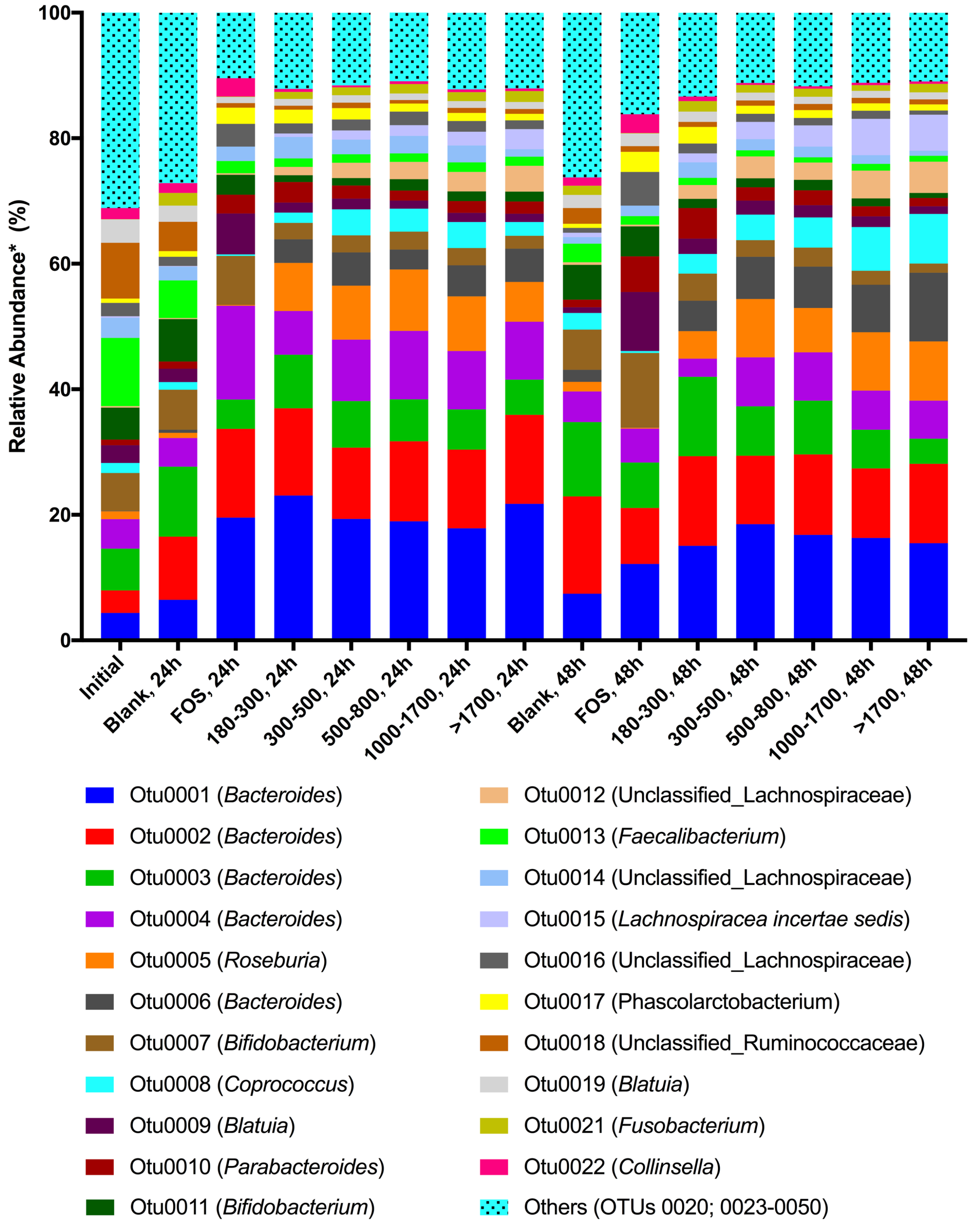

Figure S4. Relative abundances (percentage of sequences) based on the top 50 OTUs in each sample. The top 50 OTUs account for more than 90% of the total sequences of all wheat bran treatment groups at all time points (**Fig. S7**). Error bars represent the standard error of the mean of three separate replicates. Mean values with the same letter are not significantly different (Tukey's multiple comparisons test, $p < 0.05$).

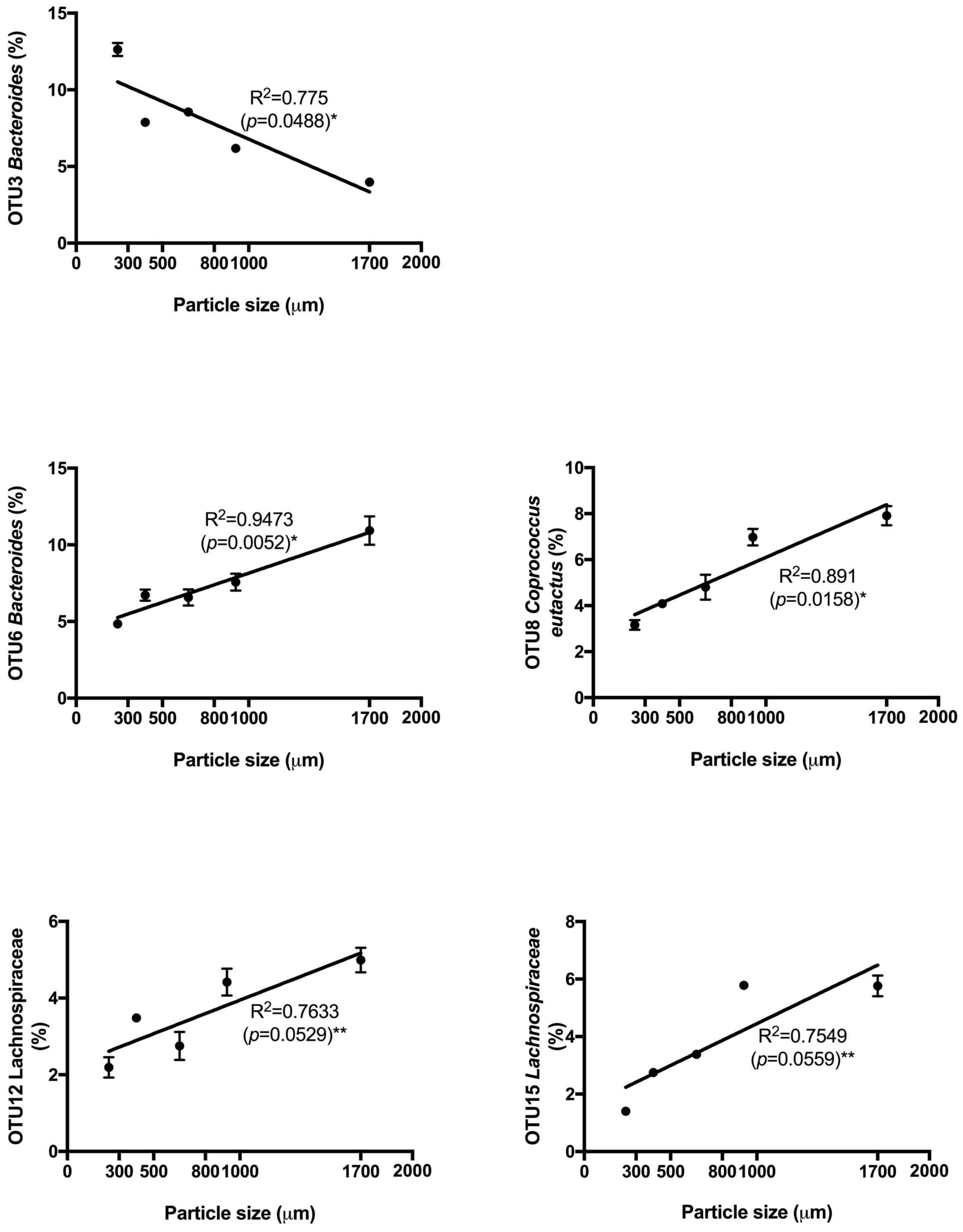

Figure S5. Regression analysis between relative abundances of specific OTUs and wheat bran particle size. Average particle size values of each wheat bran treatment groups were taken into account for regression analysis. For example, wheat bran particles between 180-300 μm in size were considered as 240 [(180+300) ÷ 2] μm for the regression analysis. The sizes of brans having >1700 μm particle size was considered as 1700 μm. * Statistically significant at α=0.05. ** Statistically significant at α=0.10.

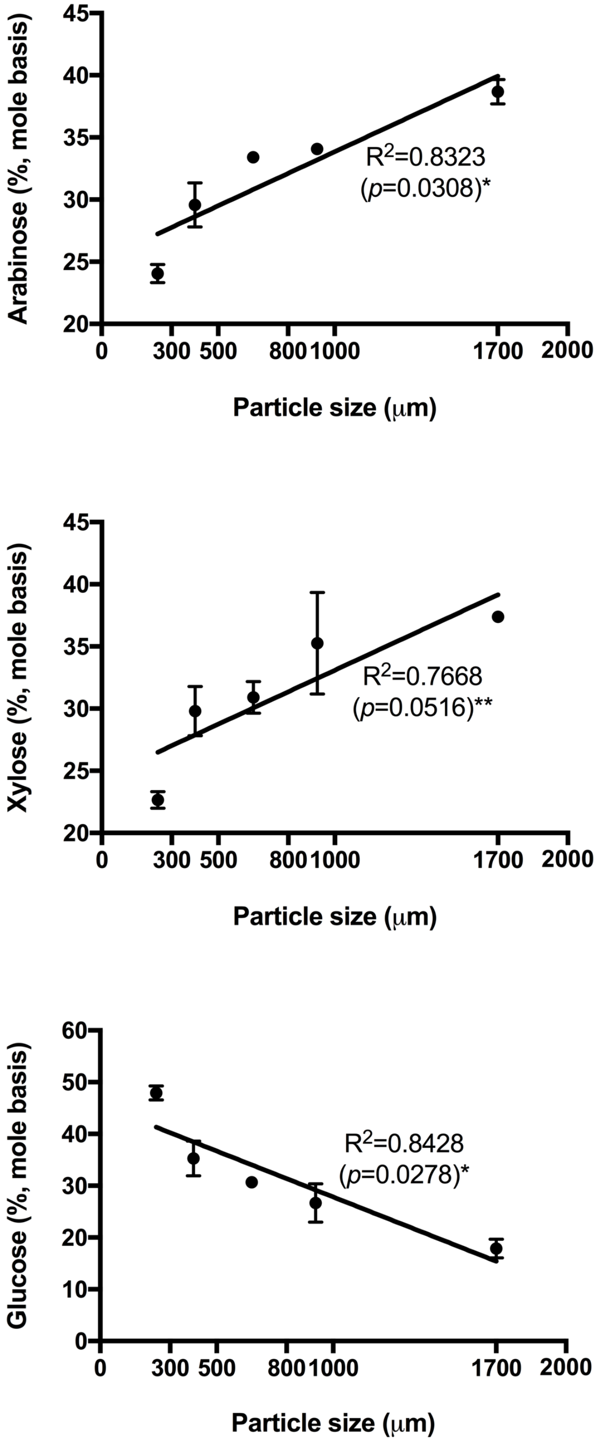

Figure S6. Regression analysis between xylose, arabinose and glucose contents and wheat bran particle size. Average particle size values of each wheat bran treatment groups were taken into account for regression analysis For example, wheat bran particles between 180-300 μm in size were considered as 240 [(180+300) ÷ 2] μm for the regression analysis. The sizes of brans having >1700 μm particle size were considered as 1700 μm. * Statistically significant at α=0.05. ** Statistically significant at α=0.10.

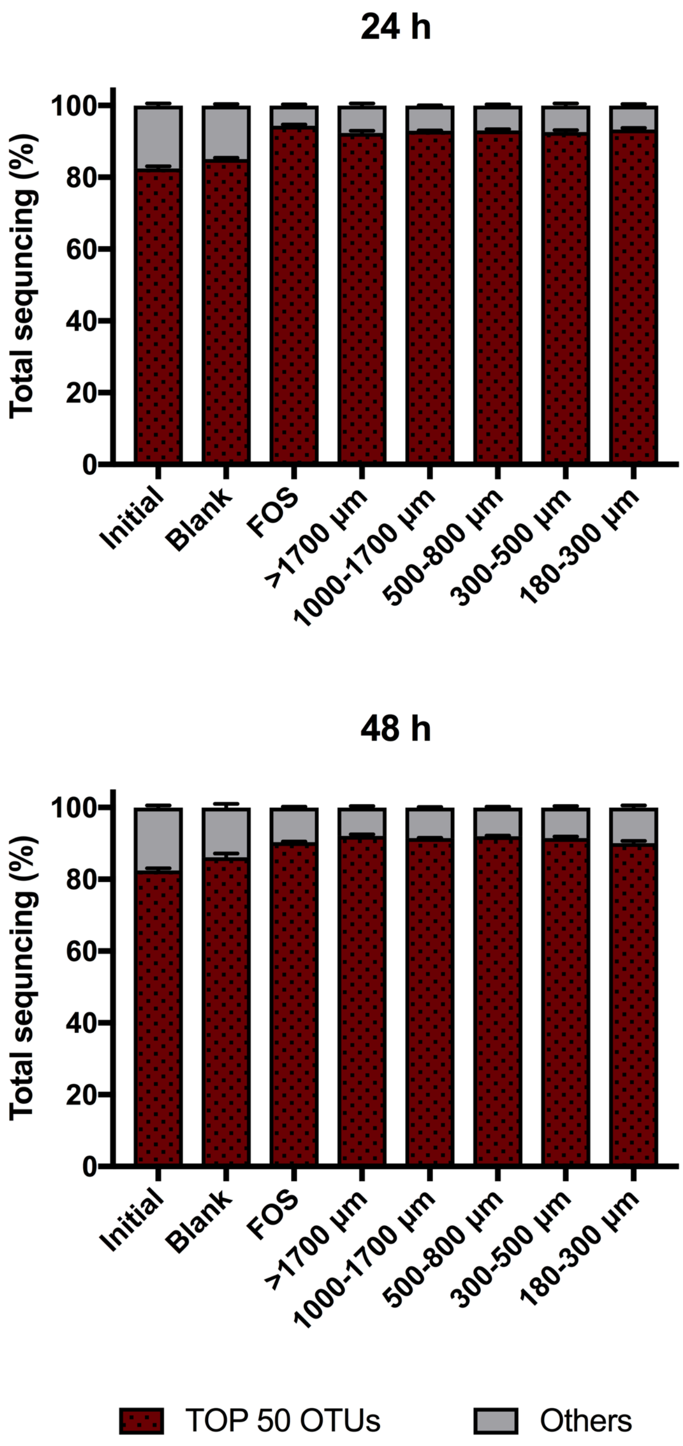

Figure S7. Relative abundances of top 50 OTUs with respect to total read counts.

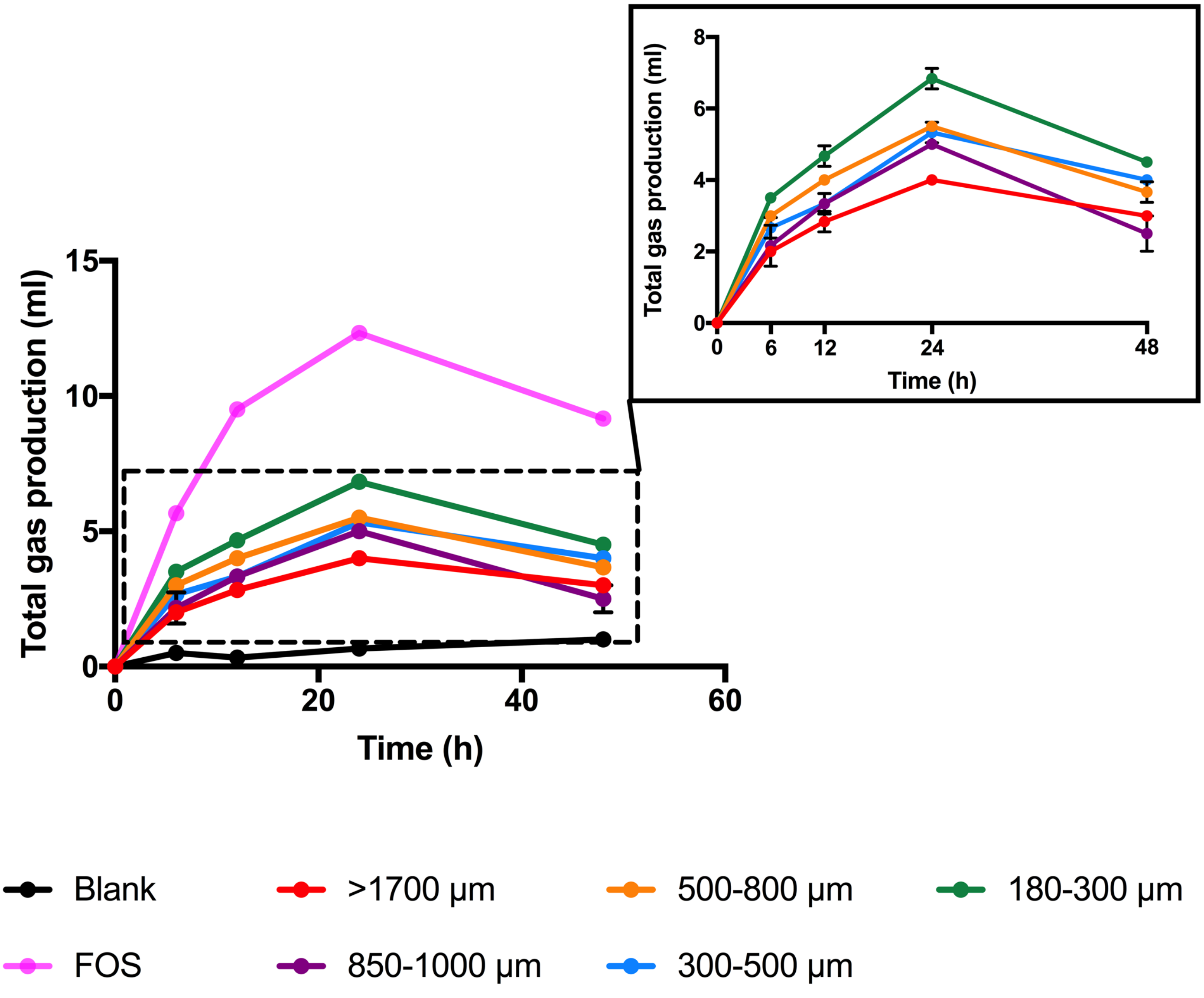

Figure S8. Total gas production by fecal microbiota through *in vitro* fermentation of different wheat bran size fractions. Error bars represent the standard error of the mean of three separate replicates.
